# Birds in the Himalayas: What drives beta diversity patterns along an elevational gradient?

**DOI:** 10.1002/ece3.4622

**Published:** 2018-11-08

**Authors:** Yiming Hu, Zhifeng Ding, Zhigang Jiang, Qing Quan, Keji Guo, Liqiao Tian, Huijian Hu, Luke Gibson

**Affiliations:** ^1^ School of Environmental Science and Engineering Southern University of Science and Technology Shenzhen China; ^2^ State Key Laboratory of Information Engineering in Surveying, Mapping and Remote Sensing Wuhan University Wuhan China; ^3^ Guangdong Key Laboratory of Animal Conservation and Resource Utilization, Guangdong Public Laboratory of Wild Animal Conservation and Utilization Guangdong Institute of Applied Biological Resources Guangzhou China; ^4^ Institute of Zoology Chinese Academy of Sciences Beijing China; ^5^ University of Chinese Academy of Sciences Beijing China; ^6^ Central South Forest Inventory and Planning Institute of State Forestry Administration Changsha China

**Keywords:** biodiversity patterns, birds, environmental factors, Gyirong Valley, mid‐domain effect, spatial factors, species turnover

## Abstract

Beta diversity patterns along elevational gradients have become a hot topic in the study of biogeography and can help illuminate the processes structuring mountain ecosystems. Although elevational species richness patterns have been well documented, there remains much uncertainty over the causes of beta diversity patterns across elevational gradients. We conducted bird surveys and obtained high‐resolution climatic data along an elevational gradient in Gyirong Valley in the central Himalayas, China, between 1,800 and 5,400 m elevation. In total, we recorded 182 bird species (including 169 breeding birds). We simulated beta diversity patterns with the mid‐domain effect (MDE) null model and conducted distance‐based redundancy analyses (db‐RDA) to relate beta diversity to dispersal limitations, spatial constraints, habitat complexity, contemporary climate, and historical climate. Mantel tests and variation partitioning were employed to identify the magnitude of independent statistical associations of environmental factors with beta diversity. Patterns of empirical and simulated beta diversity were both hump‐shaped, peaking at intermediate elevations. The db‐RDA indicated that beta diversity was correlated with changes in spatially structured environmental factors, especially with contemporary climate and habitat complexity. Mantel tests and variation partitioning also suggested that climate dissimilarity was the major independent correlate of beta diversity. The random community structure and spatial constraints may also contribute to the overall hump‐shaped pattern. Beta diversity of bird communities in Gyirong Valley could be explained by the combination of different factors but is mainly shaped by the spatially structured environmental factors, especially contemporary climate.

## 1 INTRODUCTION

Uncovering the mechanisms responsible for variation in biodiversity across space and time remains one of the central aims of ecology. The phenomenon that animal communities change along spatial or environmental gradients (e.g., from low to high elevations or from dry to moist habitats) was named “beta diversity” and defined as the “extent of species replacement or biotic change along environmental gradients” (Whittaker, 1975). Beta diversity patterns along elevational gradients, which can illuminate the processes structuring mountain ecosystems, have become a hot topic in the study of biogeography (Plants: Kitayama, 1992; Lieberman, Lieberman, Peralta, & Hartshorn, 1996; Vazquez & Givnish, 1998; Paudel & Vetaas, 2014. Moths: Brehm, Homeier, & Fiedler, 2003. Birds: Navarro, 1992; Young, DeRosier, & Powell, 1998; Blake & Loiselle, 2000; Jankowski, Ciecka, Meyer, & Rabenold, 2009). Most recent studies on the subject have sought to answer one principal question: What make ecological communities dissimilar across spatial or environmental gradients (Anderson et al., 2011)?

Although elevational species richness patterns have been well documented (Rahbek, 1995), there remains much uncertainty over the patterns of beta diversity across elevational gradients (McCain & Beck, 2016). McCain and Beck (2016) argued that elevational beta diversity patterns were changeable and difficult to use as a general explanation of elevational richness patterns. However, the highest beta diversity frequently appears at intermediate elevations, commonly accompanied by high biodiversity or associated with ecotones (Clements, 1916; Levanoni, Levin, Pe'er, Turbe, & Kark, 2011; Mena & Vazquez‐Dominguez, 2005; Whittaker, 1975).

Currently, several contemporary mechanisms have been recognized to influence the distribution of organisms and generate patterns of beta diversity: (a) the effect of environmental conditions (e.g., contemporary climate and habitat complexity. Nekola & White, 1999; O'Malley, 2008); (b) dispersal limitations (e.g., area, elevation, and geometric constraints. Colwell & Hurtt, 1994; Keil et al., 2012); and (c) species interactions (Cornell & Lawton, 1992; Gotelli, Graves, & Rahbek, 2010). However, historical mechanisms (e.g., historic climate and geographical barriers) should also not be excluded for explaining contemporary patterns of beta diversity (Leprieur et al., 2011). Stochastic processes (such as random community structure) are also considered to influence patterns of beta diversity (Chase, 2010; Chisholm & Pacala, 2011). Inspired by studies of elevational richness patterns, mid‐domain effect null models (MDE; Colwell & Hurtt, 1994) have been used to test whether species ranges vary individualistically or whether species cluster into structured zonal communities (Herzog, Kessler, & Bach, 2005; Koleff & Gaston, 2001; McCain & Beck, 2016; Mena & Vazquez‐Dominguez, 2005). Moreover, it has been argued that an interacting framework of different drivers rather than any single one should explain the beta diversity patterns (Baselga & Jimenez‐Valverde, 2007; Qian & Ricklefs, 2007).

The Himalaya Mountains, the greatest mountain range in the world, offer a unique environment to conduct studies on the mechanisms of beta diversity patterns and the ecological theories of species distribution. However, numerous studies in this area mainly focused on the elevational patterns of species richness (Acharya, Vetaas, & Birks, 2011; Grytnes & Vetaas, 2002; Hu et al., 2017, 2018, 2014; Joshi & Bhatt, 2015; Pan et al., 2016). Studies on beta diversity in the Himalayas have focused on plants in northwest Himalayan region (de Bello, Dolezal, Ricotta, & Klimesova, 2011; Saha, Rajwar, & Kumar, 2016) and trans‐Himalayan region (Paudel & Vetaas, 2014). To identify comprehensive beta diversity patterns, more rigorous studies in different regions for different taxa are needed.

Identifying underlying drivers of beta diversity can help to predict how species ranges or community composition shift with abiotic factors and biotic interactions (Jankowski et al., 2013; Kissling, Field, Korntheuer, Heyde, & Bohning‐Gaese, 2010). To better understand the mechanisms of beta diversity and help identify the necessary management actions toward preservation of biodiversity in the Himalayas, we examine beta diversity patterns of birds in the Himalayas by sampling continuously across an elevational gradient and using high‐resolution environmental or spatial variables to analyze diversity patterns. We test the hypotheses that (a) beta diversity is higher at intermediate elevations and related to species richness; (b) random community structure contributes to the beta diversity patterns; and (c) beta diversity is determined mainly by environmental conditions.

## 2 METHODS

### 2.1 Study area

**Figure 1:**
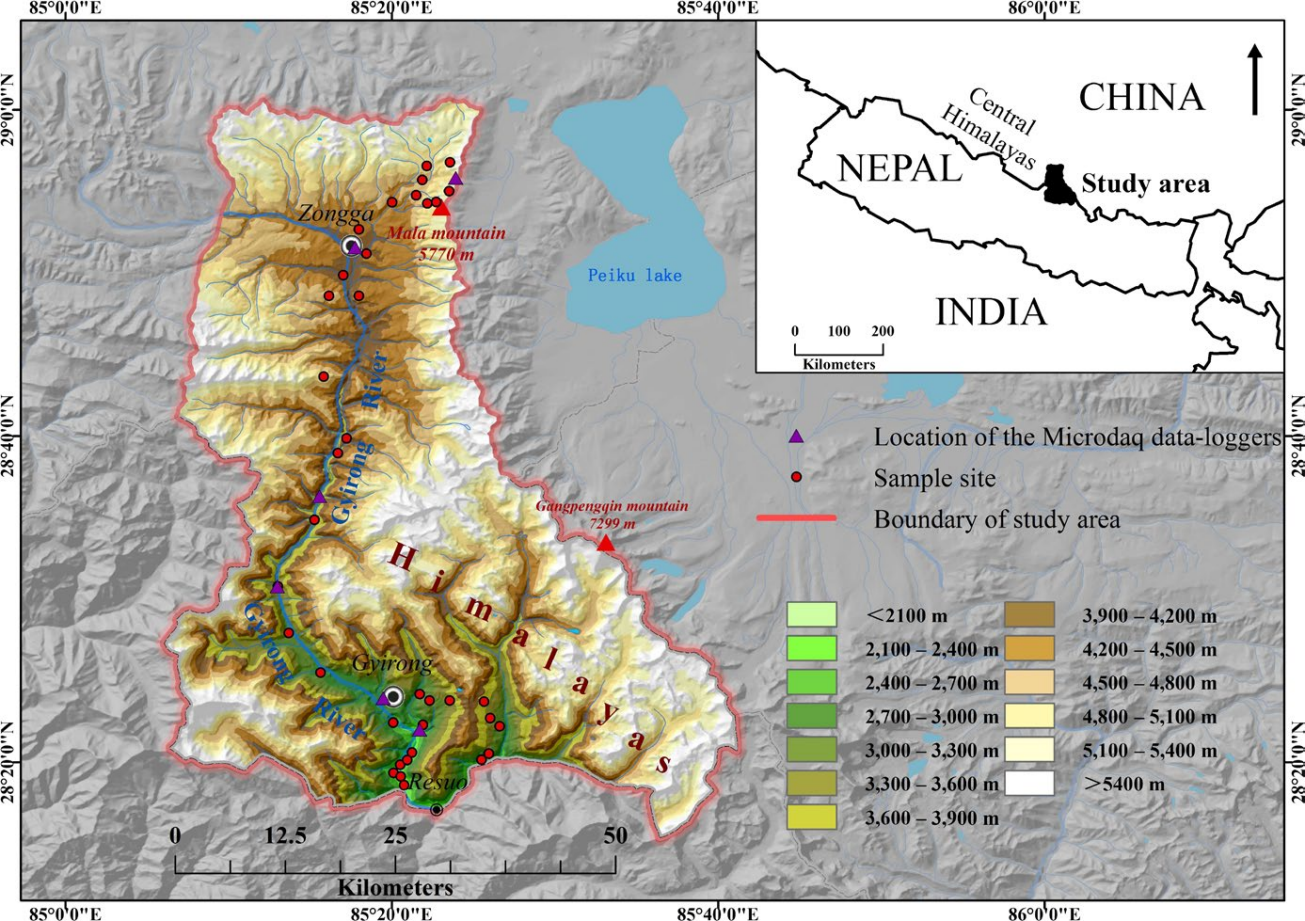
Location of the study area. The study area encompasses 12 elevational sampling bands. Sample sites display the midpoints of each transect

Gyirong Valley (28°15′‐29°0′N, 85°6′‐85°41′E) is located in the central Himalayas, China, and spans more than 4,000 m in elevation, from Resuo Village (1,700 m above sea level, asl) to the summit of Mt. Mala (5,770 m asl). The distance from the bottom of the valley to the summit of Mt. Mala is 79 km. Gyirong Valley is in a transition zone between the Oriental and Palearctic realms (Hu et al., 2014; Zhang, 2011), characterized by complicated geological structure, varied geomorphologic types, rich biodiversity, and variable mountain climate. There are five climate types in the Valley: (a) the Mountainous Subtropical Zone, (b) the Mountainous Warm Temperate Zone, (c) the Mountainous Cold Temperate Zone, (d) the Subalpine Frigid Zone, and (e) the Alpine Frigid Zone (Hu et al., 2017). The wet season approximately lasts from May to October, whereas the dry season is from November to April. The vegetation outside Gyirong Valley (alpine tundra with sparse grasses) is different from the vegetation within the valley (evergreen broadleaf forest, broadleaf mixed forest, coniferous forest, shrub and grasses). We used topography to define the study region, with the highest ridge lines and the major watercourses surrounding Gyirong Valley as the boundaries (Figure 1).

### 2.2 Sampling and sampling effort

Bird censuses were conducted from 1,800 to 5,400 m asl along the elevational gradient in Gyirong Valley. Field sampling could not be done at lower or higher elevations because the lowest elevation in this area is 1,800 m asl and the habitats above 5,400 m asl are inaccessible cliffs and glaciers. Four of the five climate zones were sampled, excluding the Alpine Frigid Zone. We divided the elevational gradient into twelve consecutive 300‐m‐wide elevational bands. There were three 300‐m elevational bands in each climate zone, and we placed three transect lines in each 300‐m elevational band to cover most of the available habitat types (for a total of 36 transect lines, see Figure 1). We standardized sampling effort across the gradient by limiting the total length of the three transect lines in each 300‐m elevational band to 7.5 km, to reduce sampling bias (Rahbek, 2005); the length of each transect was between 2 and 3 km. Field surveys were carried out between 20 min after dawn and 10:00 hours and between 16:00 hours and 20 min before sunset (local time) by the same proficient observers (J. J. Li and H. F. Cao). Bird species within 50 m of the observers were recorded during each census. The movement rate of the observers depended on bird activity. To reduce the temporal autocorrelation among transects, the transects in the different elevational bands were sampled in a random order. Replicated bird censuses were made four times during the wet season, from May to June 2012, August 2012, from September to October 2012, and from July to August 2013. We used Zheng (2011) as the taxonomic system of birds in this study. Only breeding birds (including residents and summer migrants) were used for statistical analyses, as migratory birds could cause potential bias (Pan et al., 2016).

Because of the strong movement ability of birds and the relatively small study area, we interpolated the presence of species to all elevational bands between the lowest and highest observed presences. In this study, the elevational ranges of species were then transformed by “*n* × 300” m (where “*n*” is the number of elevational bands that a species occurs). This interpolation method can reduce bias of the underestimation of species richness caused by insufficient sampling, especially in areas with high biodiversity (Wu et al., 2013). This interpolation method is widely used in elevational research and allows for comparisons with other relevant studies (Brehm, Colwell, & Kluge, 2007; Rowe, 2009; Wu et al., 2013).

We used three methods to assess how well the species diversity was sampled. Species accumulation curves have often been used to evaluate whether species diversity was sampled adequately; an adequate survey of species was assumed if the species accumulation curve reach an asymptote (Magurran & McGill, 2011). In the first method (method 1), we randomized the accumulation order of individuals 50 times by EstimateS 9.10 (Colwell, 2013; https://purl.oclc.org/estimates/) and obtained the individual‐based rarefaction curves (cumulative species number as a function of individual number). Individual‐based rarefaction is sensitive to biases in the quantification of the number of individuals per species (Gotelli & Colwell, 2001), while species richness estimation based on samples is less sensitive to this problem (Herzog et al., 2005). Thus, the second method (method 2) is sample‐based rarefaction. We used a modified version of the “m‐species‐list method” (Poulsen, Krabbe, Frølander, Hinojosa, & Quiroga, 1997) to generate small samples in each 300‐m elevational band (each m‐species list was treated as a separate sample). We combined all field surveys of each 300‐m elevational band into a master list of bird observations (each transect was surveyed separately, and records were joined according to the sequence of the survey). We then divided the master list of each 300‐m elevational band into lists of 10 species: The first 10‐species list consists of the first 10 species observed, and the second 10‐species list may include repetitions or new species compared with the first 10‐species list. We randomized the sample accumulation order 50 times using EstimateS and obtained the sample‐based rarefaction curves (cumulative species number as a function of list number).

However, it is unlikely to detect all species in natural communities even after thorough and extensive sampling. Therefore, in the third method (method 3), estimators (MMMean and Chao2) were used to compute estimated species richness. For species‐rich bird data sets, MMMean was the least biased, but MMMean could not reflect the statistically variance (Herzog, Kessler, & Cahill, 2002). Chao2 could be used as supplementary, as it is the least biased estimates of species richness for small numbers of samples (Colwell & Hurtt, 1994). The MMMean and Chao2 statistic of each 300‐m elevational band were obtained from the sample‐based rarefaction in EstimateS. To compare survey effort in different 300‐m elevational bands, observed richness was expressed as the proportion of the respective MMMean statistic (Herzog et al., 2005).

### 2.3 Measuring beta diversity

The Simpson dissimilarity index (Simpson, 1943), which reflects species compositional differences (or describes spatial turnover) without the influence of richness gradients (Leprieur et al., 2011), has been used as a measurement of beta diversity (or turnover) in elevational studies (Lennon, Koleff, Greenwood, & Gaston, 2001; McCain & Beck, 2016; Mena & Vazquez‐Dominguez, 2005). The index is defined as:

(1) $$\beta_{\mathrm{Sim}}=\frac{\min(b,c)}{a+\min(b,c)},$$

where *a* is the number of species shared by the two elevational bands, and *b* and *c* are the number of species that only appear in each of the different elevational bands. In this study, we use the *β*_sim_ between all pairs of 300‐m elevational bands to reflect the change in species composition along the elevational gradient in Gyirong Valley.

### 2.4 Environmental factors

We collected data related to dispersal limitations, geometric constraints, habitat complexity, contemporary climate, and historical climate for Gyirong Valley. Each of the environmental factors is described in detail below:

#### 2.4.1 Area and MDE

The planimetric area of each 300‐m elevational band was calculated in ArcGIS 10.4 (ESRI, Redlands, CA, USA) using 30‐m digital elevation model (DEM). The DEM data were acquired from the Geospatial Data Cloud website (GDC; https://www.gscloud.cn). The values of the area were much higher than the values of other variables, and then we log(*x*)‐transformed the values of area to alleviate heteroscedasticity.

We randomized (without replacement) the empirical species ranges within the bounded domains (from 1,800 to 5,400 m asl) to generate a predicted species richness (*R*_MDE_) under geometric constraints using RANGEMODEL 5 (https://purl.oclc.org/rangemodel; Colwell, 2008). *R*_MDE_ and their 95% confidence intervals were computed for each 300‐m band based on 10,000 simulations of the MDE rang‐model. We also used the Beta Simulation program (https://spot.colorado.edu/~mccainc/simulation_programs.htm; McCain & Beck, 2016), based on the MDE model bound randomizations to generate the predicted Beta diversity (*β*_MDE_) pattern. *β*_MDE_ and their 95% confidence intervals were computed for each 300‐m band based on 10,000 simulations of the Beta Simulation program.

#### 2.4.2 Contemporary climate

To catch the real characteristics of climate in such fine‐scale mountainous region, we established six mini weather stations in Gyirong Valley (2,457, 2,792, 3,368, 3,740, 4,140, and 5,230 m asl; Figure 1). Each mini weather station consists of three data loggers (HoBo Pro‐RH/Temp, HoBo Pro‐Precipitation/Temp, and HoBo Pro‐PAR) and was surrounded by fences to prevent interference from wild animals. Mean daily temperatures (MDT), precipitation (P), relative humidity, and photo‐synthetically active radiation were measured and recorded from September 2015 to July 2016. Only temperature and precipitation data which show more consistent spatial structured signals were used as the climatic factors in this study. Temperature data were recorded every 10 min and were averaged afterward to 1 hr to minimize the impact of possible outliers from September 2015 to July 2016 (Brehm et al., 2007). Precipitation data were accumulated also from September 2015 to July 2016. Then, the MDT and P recorded by the mini weather stations were extrapolated to all elevational bands in the study area using ArcGIS 10.4.

#### 2.4.3 Habitat complexity

We combined a 30‐m DEM and the 300‐m GlobCover landcover data of the study to obtain the landcover type in each 300‐m elevational band. We extracted the cells of the landcover raster data that correspond to the areas defined by the 30‐m DEM for each 300‐m elevational band by using the extract by mask tool in ArcGIS 10.4. We set the cell size of the result as the inputted DEM data (30‐m). Excluding the artificial areas, 21 landcover types are defined following the United Nations Land Cover Classification System (LCCS). The landcover data were from Globcover2009 (https://www.gscloud.cn/, data were accessed in 25th October 2015). We calculated the habitat heterogeneity (HH) using the Shannon diversity index.

Plant species richness represented structural complexity of the habitat (Hu, et al., 2017). To acquire plant community data in Gyirong Valley, field surveys of plants were conducted in three 20 × 20 m‐quadrats in each 300‐m elevational band. The quadrats were established alongside the transect lines. The sites selected for the plant sampling quadrats have typical representative of the vegetation communities of each 300‐m elevational band. Most of the vascular plants were measured (height, frequency, abundance, coverage, and diameter at breast height for trees), tagged, and identified. Some plant species difficult to identify were identified after the field surveys based on herbarium collections. Plant species richness (PR) of each elevational band was calculated as a habitat variable.

#### 2.4.4 Historical climate

The Quaternary Period covers the last 1.8 million years, characterized with frequent glacial advances and retreats (Leprieur et al., 2011). Two paleoclimatic variables which related to Quaternary climate stability were quantified to describe Quaternary climate stability. We extracted the annual temperature and precipitation during the Last Glacial Maximum (LGM, about 22,000 years ago) from three Global Climate Models (GCMs), named CCSM4, MIROC‐ESM, and MPI‐ESM‐P by ArcGIS 10.4 (data from the WorldClim ‐ Global Climate Data version 1.4, https://www.worldclim.org/paleo-climate1). We calculated the change in mean annual temperature and precipitation between the present and the LGM for each GCM and averaged the variation among the three GCMs (Jansson & Davies, 2008). Temperature and precipitation change between present and the LGM abbreviated as TC and PC.

Eight environmental factors were obtained for each 300‐m elevational band in Gyirong Valley, and these factors can be divided into several subsets: (a) spatial constraints (area and MDE) and (b) spatial structured environmental factors (MDT, P for contemporary climate; HH and PR for habitat complexity; TC and PC for historical climate).

### 2.5 Statistical analysis

Polynomial regression analyses were performed to assess the form of elevational patterns of *β*_sim_. We compared *β*_sim_ within the two adjacent 300‐m elevational bands with the combined species richness of those two elevational bands using linear regressions. We also compared *β*_sim_ with *β*_MDE_ to test whether random species community structure contribute to the beta diversity patterns by using linear regressions and assessing whether the elevational gradient had any *β*_sim_ outside the confidence intervals of the MDE model.

To be consistent, we preserved the same dissimilarity measure (*β*_sim_) used in the remaining statistical analyses. We conducted distance‐based redundancy analysis (db‐RDA) to explain the beta diversity by relating the bird species composition to the eight environmental factors. A site × species matrix and a site × variables (environmental factors) matrix were used in db‐RDA. Two subsequent approaches were conducted to search for parsimony and reduce the possible strong linear dependencies (collinearity) among the predictor variables. In the first approach, we used Pearson correlation to examine the relationships among the eight environmental factors and excluded the highly correlated factors based on the correlation coefficients. MDT were highly correlated with P (*r* = 0.916, *p* < 0.001) and Area (*r *= −0.944, *p* < 0.001); HH were highly correlated with MDE (*r* = 0.957, *p* < 0.001); and PC were highly correlated with Area (*r *= −0.871, *p* < 0.001) and TC (*r* = 0.851, *p* < 0.001; Supporting information Appendix S1: Table S1). After removing those highly correlated factors (MDT, HH, and PC), only P, PR, Area, MDE, and Ta would be tested in a new db‐RDA model. In the second approach, we used forward selection to select the best models based on the permutational *p* values, and on AIC value in the db‐RDA with all eight factors. However, collinearity among the predictor variables cannot be confidently resolved with empirical data sets (most commonly, collinearity is intrinsic) especially in such fine‐scale sample size. Thus, we compared the results of the three db‐RDA models to better understand the correlates of assemblage variation among the environmental factors.

To analyze variation in beta diversity, Mantel tests were conducted to explain the correlation between species dissimilarity and dissimilarity of the variable subsets. We quantified the dissimilarity in species (*β*_sim_) and dissimilarity in four variable subsets (variable‐subset.diff) between all pairs of 300‐m elevational bands and arranged the dissimilarities into sites × sites triangular “dissimilarity” matrixes (Keil et al., 2012; Winter et al., 2010). The *β*_sim_ matrix describes dissimilarity in species composition in these elevational bands. For the variable‐subset.diff, we firstly standardized and centered the variable‐subset data and then rearranged them back to the sites × values matrix; based on these sites × values matrix, we calculated matrices of Euclidean distances between all pairs of 300‐m elevational bands for spatial constraints (Spatial.diff), contemporary climate (Climate.diff), habitat complexity (Habitat.diff), and historical climate (Paleoclimatic.diff).

To assess the independent effects of different variable subsets in explaining beta diversity, we performed variance partitioning with a set of db‐RDA ordinations and partitioned the overall explained variance into components of independent and joint effects for different variable subsets.

Polynomial regression analyses were performed in PAST 3.0 (Hammer, Harper, & Ryan, 2001; https://folk.uio.no/ohammer/past/), and the other statistical analyses were performed in R 3.4.3 (R Development Core Team, 2017.) with Vegan package (Oksanen et al., 2007).

## 3 RESULTS

### 3.1 Fauna and sampling effort

**Figure 2:**
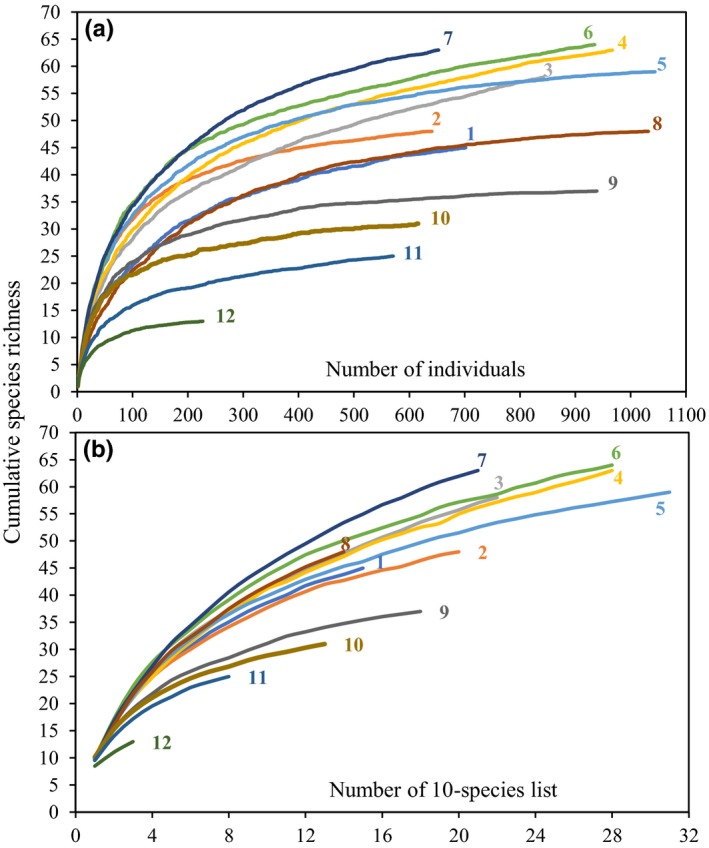
Individual‐based (a) and sample‐based (b) rarefaction curves for bird data sets for each elevational band in Gyirong Valley. The numbers from 1 to 12 represent the twelve 300‐m elevational bands from 1,800 to 5,400 m asl; for example, “1” is the 300‐m elevational band ranging from 1,800 m to 2,100 m asl. Accumulation order of all curves was randomized 50 times

We recorded a total of 182 bird species (belonging to 12 orders, 43 families, and 105 genera), including 169 breeding bird species (belonging to 11 orders, 41 families, and 100 genera; Supporting information Appendix S1: Table S2). All individual‐based rarefaction curves for each 300‐m elevational band reached a plateau or an asymptote (Figure 2a). The sample‐based rarefaction curves were steeper compared to the individual‐based curves, but most still reached an asymptote (except the 12th elevational band ranging from 5,100 to 5,400 m asl, presumably caused by the limited numbers of species or 10‐species lists in this band; Figure 2b; Supporting information Appendix S1: Fig. S1). Although empirical species richness values were lower than the estimators (MMMean and Chao2), the survey effort (observed richness/MMMean) of each 300‐m elevational band was still higher than 70% (Table 1). The rarefaction curves and survey effort indicate that the sampling effort within these 300‐m elevational bands was sufficient. Moreover, the elevational richness patterns obtained by all approaches are highly correlated (Observed richness vs. MMMean: *r* = 0.984, *p* < 0.001; Observed richness vs. Chao2: *r* = 0.911, *p* < 0.001; Interpolated richness vs. Observed richness: *r* = 0.987, *p* < 0.001; Interpolated richness vs. MMMean: *r* = 0.960, *p* < 0.001; Interpolated richness vs. Chao2: *r* = 0.938, *p* < 0.001), and as such, we conclude that our data processing approaches were appropriate in this study and the richness data can reflect the bird species richness pattern along the elevational gradient in Gyirong Valley.

**Table 1:** Observed and estimated species richness in each elevational band in Gyirong Valley

Elevation (m)	Lists	Individuals per list	Total individuals	Observed richness	Interpolated richness	MMMean statistic	Chao2 statistic	Survey effort (%)
1,800–2,100	15	46.80 ± 13.48	702	45	45	59.19	55.86 ± 7.15	76.03
2,100–2,400	20	32.05 ± 12.42	641	48	53	58.8	68.27 ± 13.84	81.63
2,400–2,700	22	38.27 ± 35.31	842	58	74	72.01	117.66 ± 36.26	80.54
2,700–3,000	28	34.54 ± 20.75	967	63	79	75.61	93.13 ± 16.32	83.32
3,000–3,300	31	33.68 ± 13.73	1,044	59	73	68.18	78.60 ± 12.37	86.54
3,300–3,600	28	33.39 ± 21.13	935	64	74	78.1	94.38 ± 18.38	81.95
3,600–3,900	21	31.10 ± 15.01	653	63	71	84.64	85.89 ± 12.13	74.43
3,900–4,200	14	73.71 ± 70.15	1,032	48	57	67.39	61.97 ± 8.44	71.23
4,200–4,500	18	49.89 ± 28.36	939	37	41	43.29	44.87 ± 6.56	85.47
4,500–4,800	13	47.38 ± 34.14	616	31	33	36.61	43.46 ± 11.54	84.68
4,800–5,100	8	71.38 ± 38.45	571	25	26	32.22	32.09 ± 6.28	77.59
5,100–5,400	3	75.67 ± 31.12	227	13	13	16.81	25.00 ± 15.87	77.33

### 3.2 Elevational patterns of environmental factors

**Figure 3:**
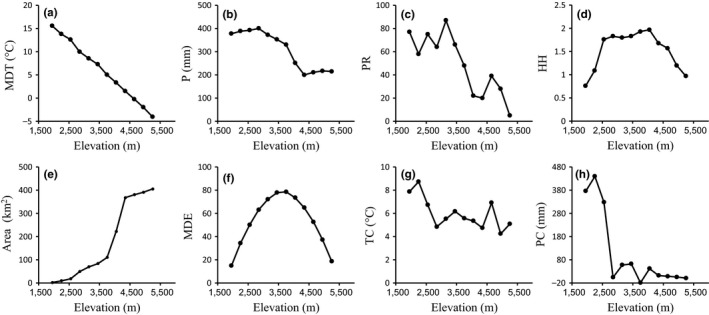
Environmental patterns across elevation in Gyirong Valley: (a) mean daily temperature, (b) precipitation, (c) plant species richness, (d) habitat heterogeneity, (e) area, (f) mid‐domain effect, (g) temperature change between present and the LGM, and (h) precipitation change between present and the LGM

Temperature (MDT) decreases monotonically with elevation (Figure 3a), whereas precipitation decreased monotonically with elevation from 2,850 to 4,350 m asl, with stable plateaus at both low and high elevations (Figure 3b). PR decreased with fluctuations and peaked at 3,000–3,300 m asl (Figure 3c). HH showed an approximate hump‐shaped pattern (Figure 3d). Area increases monotonically with elevation (Figure 3e), and MDE showed a standard hump‐shaped pattern (Figure 3f). TC and PC both peaked at low elevation (2,100–2,400 m asl) and decreased with elevation (Figure 3g, h).

### 3.3 Diversity patterns of birds

**Figure 4:**
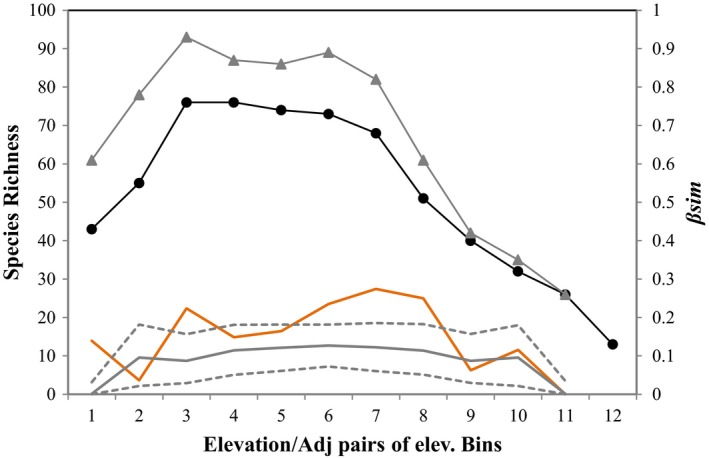
Empirical beta diversity patterns (orange line) and beta diversity patterns predicted by the MDE model (gray line) along elevational gradients (adjacent pairs of elevation bins) with the upper and lower 95% confidence interval simulation limits (gray dashed line); species richness of 300‐m elevational bands (black line with dots) and richness of those two adjacent bands combined (gray line with triangle) are also shown in the figure. The numerals of the *x*‐axis represent the twelve 300‐m elevational bands. For example, “1” refers to the first elevational band, 1,800–2,100 m asl

Species richness showed a hump‐shaped pattern along the elevational gradient in Gyirong Valley, with a peak at 2,400–3,000 m asl (76 species, Figure 4). Qualitatively, the elevational patterns of *β*_sim_ between adjacent 300‐m elevational bands and combined species richness of those pairs of adjacent bands both peaked at intermediate elevations (with a second peak; Table 2). Quantitatively, the elevational pattern of *β*_sim_ was hump‐shaped (*r* ^2^_first‐order_ = 0.0334, *p* > 0.05; *r* ^2^_second‐order_ = 0.536, *p* < 0.05; *r* ^2^_third‐order_ = 0.606, *p* > 0.05) and the correlation between *β*_sim_ and species richness was significant (*r* ^2^ = 0.513, *p < *0.01).

**Table 2:** Species richness and turnover in each elevational band in Gyirong Valley

No. of Elev. band	Elevation (m)	Richness	*β*_sim_	*β*_MDE_ ± *SD*
1&2	1,800–2,400	61	0.1395	0.001094 ± 0.0067
2&3	2,100–2,700	78	0.03636	0.09535 ± 0.041
3&4	2,400–3,000	93	0.2237	0.08711 ± 0.034
4&5	2,700–3,300	87	0.1486	0.1142 ± 0.034
5&6	3,000–3,600	86	0.1644	0.1211 ± 0.031
6&7	3,300–3,900	89	0.2353	0.1268 ± 0.028
7&8	3,600–4,200	82	0.2745	0.1222 ± 0.032
8&9	3,900–4,500	61	0.2500	0.1138 ± 0.033
9&10	4,200–4,800	42	0.06250	0.08687 ± 0.033
10&11	4,500–5,100	35	0.1154	0.09536 ± 0.041
11&12	4,800–5,400	26	0.000	0.001264 ± 0.0072

The elevational pattern of *β*_MDE_ predicted by the MDE model was also hump‐shaped, peaking at 3,300–3,900 m asl (Figure 4 and Table 2; *r* ^2^_first‐order_ < 0.01, *p* > 0.05; *r* ^2^_second‐order_ = 0.837, *p* < 0.01; *r* ^2^_third‐order_ = 0.837, *p* < 0.01). The correlation between the simulated *β*_MDE_ pattern and empirical *β*_sim_ pattern was moderate but significant (*r* ^2^ = 0.446, *p* < 0.05). Most of the empirical *β*_sim_ values were higher than the simulated values with five empirical *β*_sim_ points (two peaks) falling outside the 95% confidence intervals of the MDE model (Figure 4).

### 3.4 Factors explaining beta diversity patterns

**Figure 5:**
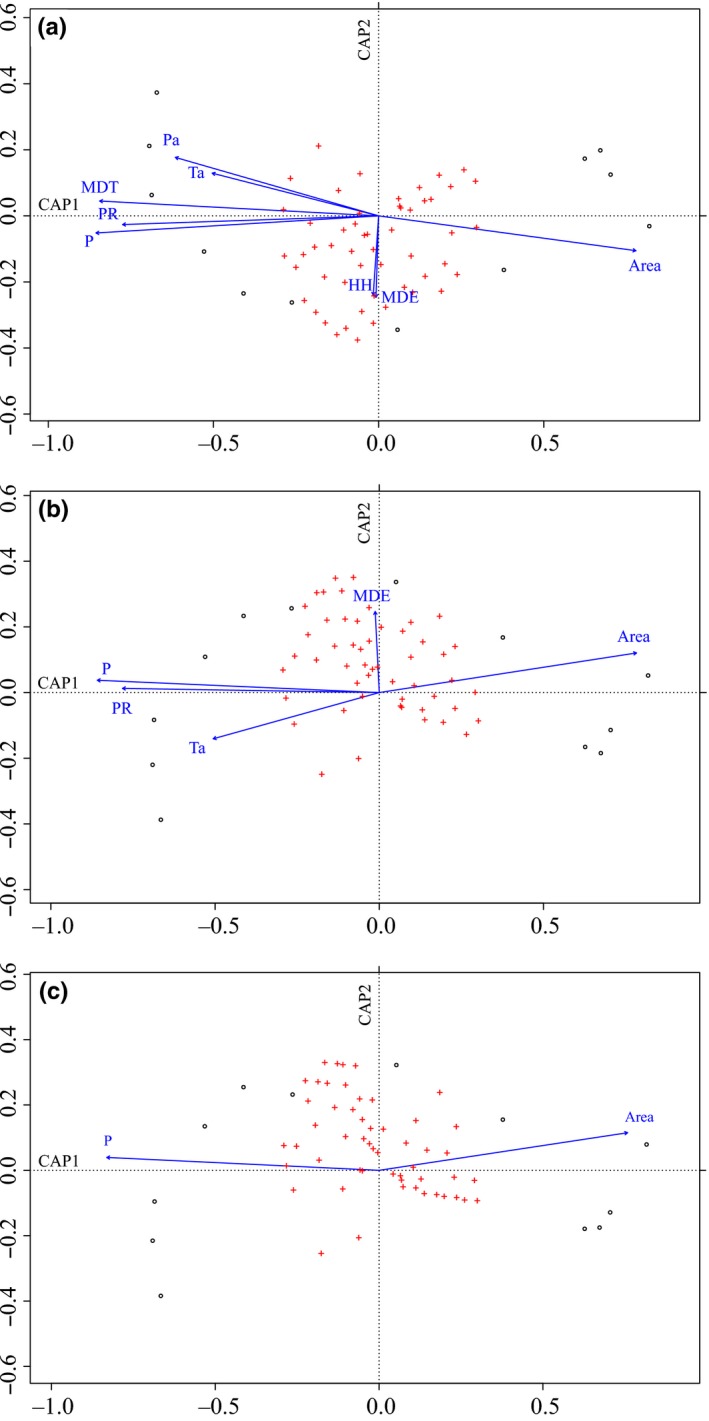
Plots of points (red cross: 169 species; circle: 12 elevation bands) and environmental variables (lines) from distance‐based redundancy analyses with (a) eight variables, (b) five variables, and (c) two variables (using Weighted Average scores). MDT: mean daily temperature; P: precipitation; PR: plant species richness; HH: habitat heterogeneity; MDE: mid‐domain effect; TC and PC: temperature and precipitation change between present and the LGM. The lines represent the direction (orientation with respect to the axis) and strength (length of the line) of the correlations between environmental variables and variation in species composition

The results of the db‐RDA with all eight environmental variables (db‐RDA.all) showed that 99% of the variation in species composition across the elevational gradient in Gyirong Valley could be explained (Total Inertia Eigenvalue *λ*_Total_ = 1.977, Constrained Inertia Eigenvalue *λ*_constrained_ = 1.964). Of the eight constrained (ordination) axes, axis No. 1 (*λ*_1_ = 1.725) explained 87% of the total variation in the data set and 88% of the variation explained by the eight constrained axes; axis No. 2 (the scatter orthogonal to axis No.1, *λ*_2_ = 0.221) explained 11% of the total variation and 11% of the variation explained by constrained axes. Axis No. 1 could be understood as a climate‐habitat gradient because it correlated positively with area (area was highly correlated with elevation, *r* = 0.947, *p* < 0.01) and negatively correlated with all the other environmental factors (Figure 5a). The correlation between the variables and the constrained axis No. 1 was shown by the direction cosines of the environmental vectors and is highest for MDT (−0.990) and PR (−0.996). Axis No. 2 was most strongly correlated with MDE (−0.999) and HH (0.999), although axis No. 2 explained a small part of the variation in species composition across the elevational gradient. The length of the arrow was proportional to the correlation between ordination and environmental variable which is called the strength of the gradient (*r* ^2^). And the *r* ^2^ of all the eight variables was statistically high (*r* ^2^_MDT_ = 0.965, *p* < 0.01; *r* ^2^_P_ = 0.984, *p* < 0.01; *r* ^2^_PR_ = 0.802, *p* < 0.01; *r* ^2^_HH_ = 0.561, *p* < 0.05; *r* ^2^_Area_ = 0.913, *p* < 0.01; *r* ^2^_MDE_ = 0.593, *p* < 0.05; *r* ^2^_TC_ = 0.500, *p* < 0.05; *r* ^2^_PC_ = 0.808, *p* < 0.01).

After removing those highly correlated factors, db‐RDA with five variables (P, PR, Area, MDE, and TC; db‐RDA.select) explained 96% of the variation in species composition (*λ*_Total_ = 1.977, *λ*_constrained_ = 1.904). Axis No. 1 (*λ*_1_ = 1.703) explained 86% of the total variation in the data set and 89% of the variation explained by the five constrained axes; axis No. 2 (*λ*_2_ = 0.185) explained 9% of the total variation and 10% of the variation explained by the constrained axes (Figure 5b). The direction cosines between the environmental vectors and axis No. 1 are highest for PR, P (−0.990) and Area (0.94602). Axis No. 2 is most correlated with MDE (0.997) and TC (−0.549).

The best‐fit models produced by forward selection based on permutational *p* values and on AIC showed that most parsimonious attitude would be to settle for the db‐RDA model containing only two environmental variables: *P* and Area. However, the db‐RDA with these two factors (db‐RDA.step) still explained 94% of the variation in species composition (*λ*_Total_ = 1.977, *λ*_constrained_ = 1.863). Axis No. 1 (*λ*_1_ = 1.696) explained 86% of the total variation in the data set and 91% of the variation explained by the two constrained axes; axis No. 2 (*λ*_2_ = 0.172) explained 9% of the total variation and 9% of the variation explained by the constrained axes (Figure 5c). More details of the three db‐RDA models can be found in the Supporting information Appendix S2.

**Figure 6:**
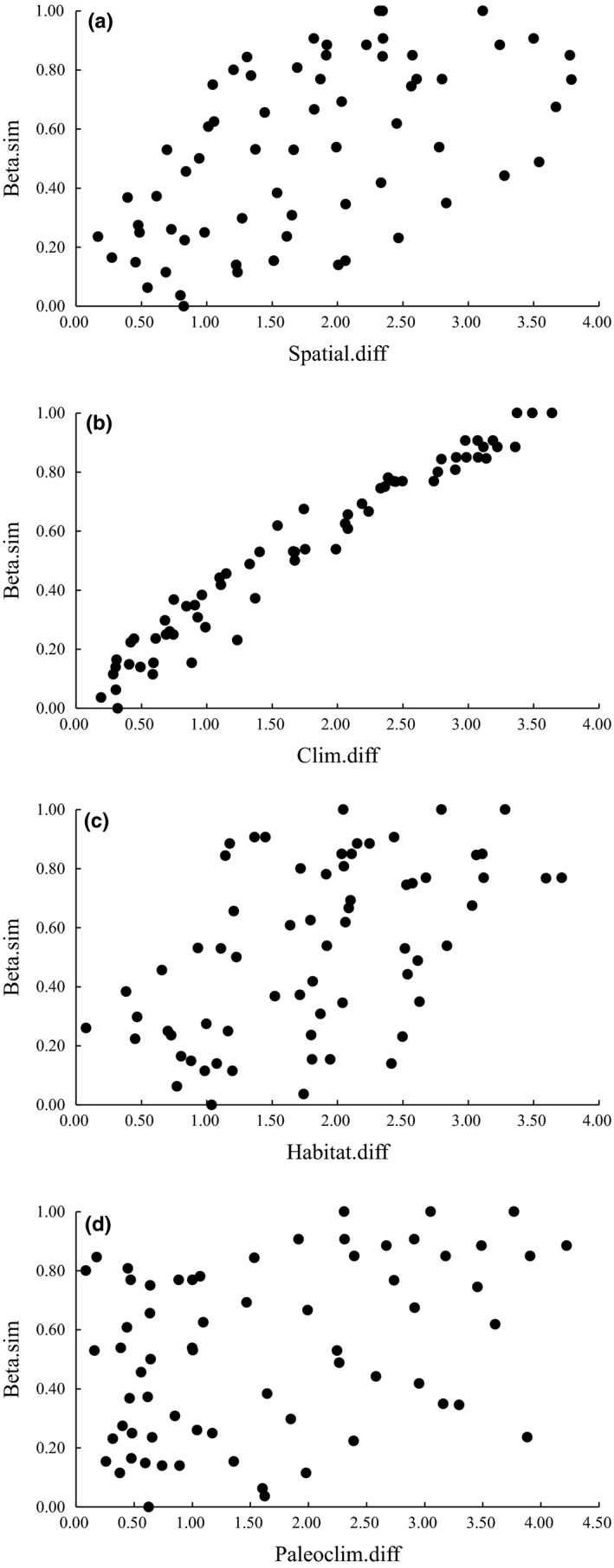
*β*_sim_ dissimilarity versus (a) dissimilarity between pure spatial factors, (b) contemporary climate dissimilarity, (c) habitat complexity dissimilarity, and (d) paleoclimate dissimilarity. (a) Mantel statistic *r* = 0.556, *p* < 0.01, 999 permutations. (b) Mantel statistic *r* = 0.977, *p* < 0.01, 999 permutations. (c) Mantel statistic *r* = 0.527, *p* < 0.01, 999 permutations. (d) Mantel statistic *r* = 0.372, *p* < 0.01, 999 permutations

Beta diversity of bird species in Gyirong Valley showed strongest significant positive correlation with contemporary climate dissimilarity (Mantel statistic *r* = 0.977, *p* < 0.01, 999 permutations; Figure 6b) and moderate significant positive correlation with dissimilarity between spatial constraints (Mantel statistic *r* = 0.556, *p* < 0.01, 999 permutations; Figure 6a) and habitat complexity dissimilarity (Mantel statistic *r* = 0.527, *p* < 0.01, 999 permutations; Figure 6c). The correlation between beta diversity and paleoclimate dissimilarity was relatively weaker but significant (Mantel statistic *r* = 0.372, *p* < 0.01, 999 permutations; Figure 6d).

**Figure 7:**
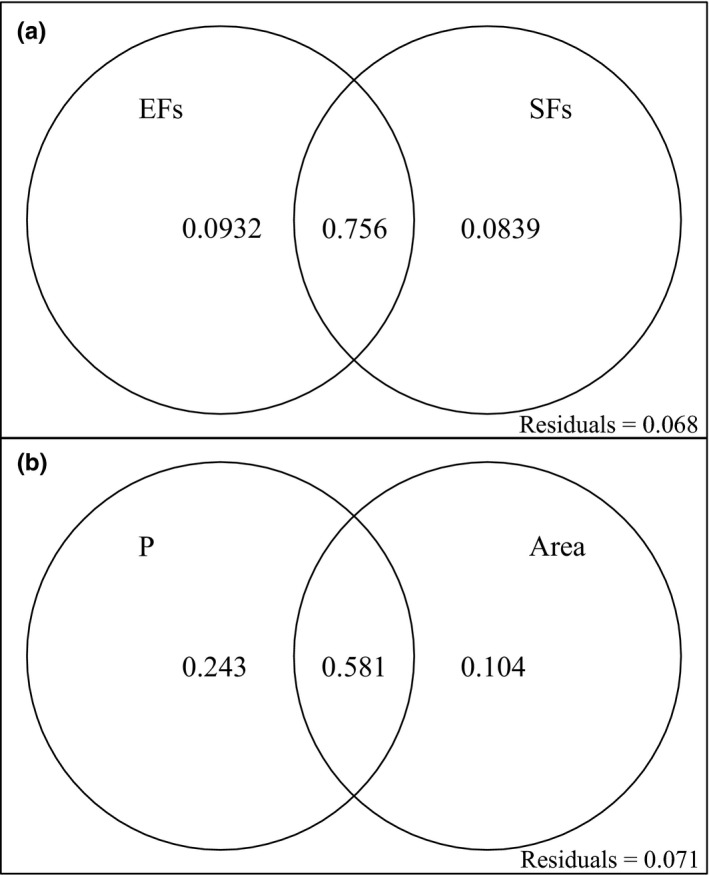
Venn diagrams of variation partitioning with a set of db‐RDA ordinations, (a) variance partitioning based db‐RDA with five variables and (b) variance partitioning based db‐RDA with two variables. EFs: spatial structured environmental factors (Precipitation, Plant species richness and temperature change between present and the LGM); SFs: spatial constraints (Area and species richness under geometric constraints); P: precipitation

We performed variance partitioning based on two db‐RDA models (db‐RDA.select and db‐RDA.step) and partitioned the overall explained variance into components of independent (*I*) and joint effects (*J*) for spatial structured environmental factors (EFs) and spatial constraints (SFs). For db‐RDA.select, the overall explained variance was partitioned into components of independent and joint effects of EF_1_ (P, PR, and TC) and SF_1_ (Area and MDE). The results of variation partitioning based on db‐RDA.select showed that EF_1_ explained more of the overall variance of species dissimilarity than SF_1_ (*I*_EF1_ = 0.0932, *I*_SF1_ = 0.0839, and *J* = 0.756; Figure 7a). For db‐RDA.step, variance was partitioned into components of independent and joint effects of P (EF_2_) and Area (SF_2_). The results of variation partitioning based on db‐RDA.step showed that P explained more of the overall variance of species dissimilarity than Area (*I*_EF2_ = 0.243, *I*_SF1_ = 0.104, and *J* = 0.581; Figure 7b).

## 4 DISCUSSION

### 4.1 Elevational patterns of beta diversity

In this study, we found that beta diversity along the elevational gradient showed a hump‐shaped pattern, peaking at intermediate elevations (and with a second peak at higher elevations, Figure 4). There is a long‐standing hypothesis that ecosystems with high beta diversity (or turnover) are accompanied by high biodiversity (Clements, 1916; Stevens, 1992; Whittaker, 1975; Wilson & Shmida, 1984), and beta diversity has been confirmed to contribute to overall species richness patterns (Buckley & Jetz, 2008; Davies, Scholtz, & Chown, 1999; Fattorini, 2014; Herzog et al., 2005; McCain & Beck, 2016; Naniwadekar & Vasudevan, 2007). Our study provides evidence to support the hypothesis that beta diversity is a driver of species richness along elevational gradients: beta diversity was consistent with species richness (*r* ^2^ = 0.626, *p* < 0.01).

Beta diversity patterns predicted by the MDE null model (*β*_MDE_) were also hump‐shaped, consistent with the empirical *β*_sim_ patterns (Figure 4). However, the predictions of mid‐domain null models were also not unified, with the predicted beta diversity (or turnover) patterns showing shallow‐unimodal patterns, bimodal patterns, and U‐shaped patterns due to the difference of method, scale, and study area (McCain & Beck, 2016). Although the predictions of the null models were inconsistent, most of the studies could not reject the null prediction (few empirical beta diversity values fell outside the 95% confidence intervals of the MDE null model predictions; Koleff & Gaston, 2001; McCain & Beck, 2016; Mena & Vazquez‐Dominguez, 2005). Only one exception rejected the random structuring assumption (Herzog et al., 2005). In our study, five (of 12) empirical *β*_sim_ points fell outside the 95% confidence intervals of the MDE null model, but with a moderate (significant) correlation between the empirical and MDE‐modeled patterns. These indicated that overall shape of the patterns may be correlated with the MDE, but that the peaks of beta diversity were not explained by the MDE model. We should note that spatial constraints and stochastic processes may play some role in shaping the beta diversity pattern for birds along the elevational gradient in Gyirong Valley.

Some studies have found that beta diversity is related to ecotones (Jankowski et al., 2009; Vazquez & Givnish, 1998). In Gyirong Valley, beta diversity peaked at 2,400–3,000 m asl (the transition zone between evergreen broadleaf forest and broadleaf mixed forest), and 3,600–4,200 m asl (the transition zone between dark coniferous forest and shrub and grass habitat). Moreover, the region from 3,100 to 4,000 m asl in the Himalaya Mounts is an ecotone between the Oriental and Palearctic regions (Hu et al., 2014). These might explain the patterns of beta diversity in Gyirong Valley.

### 4.2 Effects of environmental factors on beta diversity

In this study, results of the db‐RDA models suggested that species were distributed along a climate‐habitat gradient. Additionally, our study found high rates of beta diversity in the ecotones with more precipitation and higher temperature (at 2,400–2,700 m: MDT = 12.62°C, *p* = 392.72 mm; at 2,700–3,000 m: MDT = 10.01°C, *p* = 400.41 mm). Results of Mantel tests suggested that the spatially structured environmental factors (especially climate dissimilarity) were the strongest factors explaining beta diversity along the elevational gradient (Figure 6), and results of variation partitioning were also consistent with the Mantel test. The evidence above illustrates that beta diversity in Gyirong Valley was influenced by the spatially structured environmental factors, especially the combination of climate and habitat. However, some have argued over the relative importance of climatic factors and the importance of the resolution of climatic data (Keil et al., 2012). This should call for caution that we should use higher resolution data to cope with ecological studies in fine‐scales, especially in mountainous areas with high biodiversity, complex landscapes, and variable climate.

Researchers have found that spatially structured environmental factors (environmental filters) explained a large amount of variation in beta diversity across multiple taxa (Alard & Poudevigne, 2000; Balvanera, Lott, Segura, Siebe, & Islas, 2002; Baselga, 2008; Gaston et al., 2007; Harrison, Ross, & Lawton, 1992; Jankowski et al., 2009; Soininen, Lennon, & Hillebrand, 2007; Spencer, Schwartz, & Blaustein, 2002; Svenning, Flojgaard, & Baselga, 2011; Wolf, 1993). Nonetheless, some studies have argued that beta diversity depended mainly on geographic distance (Keil et al., 2012; Qian, Ricklefs, & White, 2005; Tuomisto, Ruokolainen, & Yli‐Halla, 2003) or on stochastic processes (Chase, 2010; Chisholm & Pacala, 2011). Although we emphasized the important influence of environmental filters on the elevational patterns of beta diversity in Gyirong Valley, the spatial constraints and stochastic processes (area and MDE) still had considerable influence.

According to previous studies, effects of environmental filtering are stronger at the local scale, while dispersal limitations play an important role at the larger regional scale (Rejmanek, 2000). In Gyirong Valley, the reason why the main driver for beta diversity patterns was environmental filtering (rather than dispersal limitations or other) may simply be that elevational bands along the elevational gradient were not sufficiently isolated, and the mobility of avian species is quite strong. The distances along the elevational gradient in Gyirong Valley (the planimetric distance from the bottom of the valley to the summit of Mt. Mala is only 79 km) might not be great enough to prevent the birds from dispersing. Still, another reason might be that the environment in Gyirong Valley is heterogeneous and variable. Habitats with different environments should harbor different sets of species, and the more different the environment, the greater the beta diversity (turnover) would be.

## 5 CONCLUSION

In general, environmental filters, habitat heterogeneity, spatial constraints, and stochastic processes influence beta diversity patterns simultaneously. The beta diversity patterns of birds in Gyirong Valley were hump‐shaped, peaking at intermediate elevations, in parallel with species richness patterns. This pattern could be explained by the combination of different factors: the overall hump‐shape might be correlated with environmental filters (playing more important roles), dispersal limitations, or stochastic processes. Further, the beta diversity peaks falling outside the confidence intervals of the MDE null model might relate to the ecotones. Climate and habitat also play important roles in shaping beta diversity patterns, and highlight the need to conserve intact habitat in this mountain area.

## CONFLICT OF INTEREST

None declared.

## AUTHOR CONTRIBUTIONS

Y.H., Z.D., and H.H. conceived the idea for this study and designed the research; Q.Q. improved the statistics method; Y.H. analyzed the data; L.G., Z.J., L.T., and K.G. developed the presentation; and Y.H. wrote the paper.

## DATA ACCESSIBILITY

The raw data have been supplied as Supplementary Files.

## References

[ece34622-bib-0001] Acharya, K. P. , Vetaas, O. R. , & Birks, H. J. B. (2011). Orchid species richness along Himalayan elevational gradients. Journal of Biogeography, 38, 1821–1833. 10.1111/j.1365-2699.2011.02511.x

[ece34622-bib-0002] Alard, D. , & Poudevigne, I. (2000). Diversity patterns in grasslands along a landscape gradient in northwestern France. Journal of Vegetation Science, 11, 287–294. 10.2307/3236809

[ece34622-bib-0003] Anderson, M. J. , Crist, T. O. , Chase, J. M. , Vellend, M. , Inouye, B. D. , Freestone, A. L. , … Swenson, N. G. (2011). Navigating the multiple meanings of beta diversity: A roadmap for the practicing ecologist. Ecology Letters, 14, 19–28. 21070562 10.1111/j.1461-0248.2010.01552.x

[ece34622-bib-0004] Balvanera, P. , Lott, E. , Segura, G. , Siebe, C. , & Islas, A. (2002). Patterns of beta‐diversity in a Mexican tropical dry forest. Journal of Vegetation Science, 13, 145–158.

[ece34622-bib-0005] Baselga, A. (2008). Determinants of species richness, endemism and turnover in European longhorn beetles. Ecography, 31, 263–271. 10.1111/j.0906-7590.2008.5335.x

[ece34622-bib-0007] Baselga, A. , & Jimenez‐Valverde, A. (2007). Environmental and geographical determinants of beta diversity of leaf beetles (Coleoptera : Chrysomelidae) in the Iberian Peninsula. Ecological Entomology, 32, 312–318. 10.1111/j.1365-2311.2007.00880.x

[ece34622-bib-0009] Blake, J. G. , & Loiselle, B. A. (2000). Diversity of birds along an elevational gradient in the Cordillera Central, Costa Rica. The Auk, 117, 663–686. 10.1642/0004-8038(2000)117[0663:DOBAAE]2.0.CO;2

[ece34622-bib-0010] Brehm, G. , Colwell, R. K. , & Kluge, J. (2007). The role of environment and mid‐domain effect on moth species richness along a tropical elevational gradient. Global Ecol Biogeogr, 16, 205–219. 10.1111/j.1466-8238.2006.00281.x

[ece34622-bib-0011] Brehm, G. , Homeier, J. , & Fiedler, K. (2003). Beta diversity of geometrid moths (Lepidoptera : Geometridae) in an Andean montane rainforest. Diversity and Distributions, 9, 351–366. 10.1046/j.1472-4642.2003.00023.x

[ece34622-bib-0012] Buckley, L. B. , & Jetz, W. (2008). Linking global turnover of species and environments. P Natl Acad Sci USA, 105, 17836–17841. 10.1073/pnas.0803524105 PMC2584760 19001274

[ece34622-bib-0014] Chase, J. M. (2010). Stochastic community assembly causes higher biodiversity in more productive environments. Science, 328, 1388–1391. 10.1126/science.1187820 20508088

[ece34622-bib-0016] Chisholm, R. A. , & Pacala, S. W. (2011). Theory predicts a rapid transition from niche‐structured to neutral biodiversity patterns across a speciation‐rate gradient. Theoretical Ecology, 4, 195–200. 10.1007/s12080-011-0113-5

[ece34622-bib-0017] Clements, F. E. (1916). Plant Succession. An analysis of the development of vegetation. Carnegie Institution of Washington Publ, 242, 339–341.

[ece34622-bib-0018] Colwell, R. K. (2008). RangeModel: Tools for exploring and assessing geometric constraints on species richness (The mid‐domain effect) along transects. Ecography, 31, 4–7. 10.1111/j.2008.0906-7590.05347.x

[ece34622-bib-0019] Colwell, R. K. , & Hurtt, G. C. (1994). Nonbiological Gradients in Species Richness and a Spurious Rapoport Effect. American Naturalist, 144, 570–595. 10.1086/285695

[ece34622-bib-0022] Colwell, R. K. (2013) EstimateS (data analysis software system and user's guide). Version 9.0. Retrieved from https://viceroy.eeb.uconn.edu/estimates/

[ece34622-bib-0023] Cornell, H. V. , & Lawton, J. H. (1992). Species interactions, local and regional processes, and limits to the richness of ecological communities—a theoretical perspective. Journal of Animal Ecology, 61, 1–12. 10.2307/5503

[ece34622-bib-0024] Davies, A. L. V. , Scholtz, C. H. , & Chown, S. L. (1999). Species turnover, community boundaries and biogeographical composition of dung beetle assemblages across an altitudinal gradient in South Africa. Journal of Biogeography, 26, 1039–1055. 10.1046/j.1365-2699.1999.00335.x

[ece34622-bib-0025] de Bello, F. , Dolezal, J. , Ricotta, C. , & Klimesova, J. (2011). Plant clonal traits, coexistence and turnover in East Ladakh, Trans‐Himalaya. Preslia, 83, 315–327.

[ece34622-bib-0026] Fattorini, S. (2014). Disentangling the effects of available area, mid‐domain constraints, and species environmental tolerance on the altitudinal distribution of tenebrionid beetles in a Mediterranean area. Biodiversity and Conservation, 23, 2545–2560. 10.1007/s10531-014-0738-y

[ece34622-bib-0027] Gaston, K. J. , Davies, R. G. , Orme, C. D. L. , Olson, V. A. , Thomas, G. H. , Ding, T. S. , … Blackburn, T. M. (2007). Spatial turnover in the global avifauna. P R Soc B, 274, 1567–1574. 10.1098/rspb.2007.0236 PMC2169276 17472910

[ece34622-bib-0028] Gotelli, N. J. , & Colwell, R. K. (2001). Quantifying biodiversity: Procedures and pitfalls in the measurement and comparison of species richness. Ecology Letters, 4, 379–391. 10.1046/j.1461-0248.2001.00230.x

[ece34622-bib-0029] Gotelli, N. J. , Graves, G. R. , & Rahbek, C. (2010). Macroecological signals of species interactions in the Danish avifauna. Proceedings of the National Academy of Sciences of the USA, 107, 5030–5035. 10.1073/pnas.0914089107 20194760 PMC2841898

[ece34622-bib-0030] Grytnes, J. A. , & Vetaas, O. R. (2002). Species richness and altitude: A comparison between null models and interpolated plant species richness along the Himalayan altitudinal gradient, Nepal. American Naturalist, 159, 294–304. 10.1086/338542 18707381

[ece34622-bib-0031] Hammer, Ø. , Harper, D. A. T. , & Ryan, P. D. (2001). PAST‐Palaeontological statistics. www.uv.es/~pardomv/pe/2001_1/past/pastprog/past.pdf, acessado em, 25(07), 2009

[ece34622-bib-0032] Harrison, S. , Ross, S. J. , & Lawton, J. H. (1992). Beta‐diversity on geographic gradients in Britain. Journal of Animal Ecology, 61, 151–158. 10.2307/5518

[ece34622-bib-0033] Herzog, S. K. , Kessler, M. , & Bach, K. (2005). The elevational gradient in Andean bird species richness at the local scale: A foothill peak and a high‐elevation plateau. Ecography, 28, 209–222. 10.1111/j.0906-7590.2005.03935.x

[ece34622-bib-0034] Herzog, S. K. , Kessler, M. , & Cahill, T. M. (2002). Estimating species richness of tropical bird communities from rapid assessment data. The Auk, 119, 749–769. 10.1642/0004-8038(2002)119[0749:ESROTB]2.0.CO;2

[ece34622-bib-0035] Hu, Y. M. , Jin, K. , Huang, Z. W. , Ding, Z. F. , Liang, J. C. , Pan, X. Y. , … Jiang, Z. G. (2017). Elevational patterns of non‐volant small mammal species richness in Gyirong Valley, Central Himalaya: Evaluating multiple spatial and environmental drivers. Journal of Biogeography, 44, 2764–2777. 10.1111/jbi.13102.

[ece34622-bib-0036] Hu, Y. M. , Liang, J. C. , Jin, K. , Ding, Z. F. , Zhou, Z. X. , Hu, H. J. , & Jiang, Z. G. (2018). The elevational patterns of mammalian richness in the Himalayas. Biodiversity Science, 26, 191–201.

[ece34622-bib-0037] Hu, Y. M. , Yao, Z. J. , Huang, Z. W. , Tian, Y. , Li, H. B. , Pu, Q. , … Hu, H. J. (2014). Mammalian fauna and its vertical changes in Mt. Qomolangma National Nature Reserve, Tibet, China. Acta Theriologica Sinica, 34, 28–37.

[ece34622-bib-0038] Jankowski, J. E. , Ciecka, A. L. , Meyer, N. Y. , & Rabenold, K. N. (2009). Beta diversity along environmental gradients: Implications of habitat specialization in tropical montane landscapes. Journal of Animal Ecology, 78, 315–327. 10.1111/j.1365-2656.2008.01487.x 19040686

[ece34622-bib-0039] Jankowski, J. E. , Merkord, C. L. , Rios, W. F. , Cabrera, K. G. , Revilla, N. S. , & Silman, M. R. (2013). The relationship of tropical bird communities to tree species composition and vegetation structure along an andean elevational gradient. Journal of Biogeography, 40, 950–962. 10.1111/jbi.12041

[ece34622-bib-0040] Jansson, R. , & Davies, T. J. (2008). Global variation in diversification rates of flowering plants: Energy vs. climate change. Ecology Letters, 11, 173–183. 10.1111/j.1461-0248.2007.01138.x 18070100

[ece34622-bib-0041] Joshi, K. , & Bhatt, D. (2015). Avian species distribution along elevation at Doon Valley (foot hills of western Himalayas), Uttarakhand, and its association with vegetation structure. Journal of Asia‐Pacific Biodiversity, 8, 158–167. 10.1016/j.japb.2015.04.002

[ece34622-bib-0042] Keil, P. , Schweiger, O. , Kuhn, I. , Kunin, W. E. , Kuussaari, M. , Settele, J. , … Storch, D. (2012). Patterns of beta diversity in Europe: The role of climate, land cover and distance across scales. Journal of Biogeography, 39, 1473–1486. 10.1111/j.1365-2699.2012.02701.x

[ece34622-bib-0043] Kissling, W. D. , Field, R. , Korntheuer, H. , Heyde, U. , & Bohning‐Gaese, K. (2010). Woody plants and the prediction of climate‐change impacts on bird diversity. Philosophical Transactions of the Royal Society B: Biological Sciences, 365, 2035–2045. 10.1098/rstb.2010.0008 PMC2880125 20513712

[ece34622-bib-0044] Kitayama, K. (1992). An Altitudinal Transect Study of the Vegetation on Mount Kinabalu, Borneo. Vegetatio, 102, 149–171. 10.1007/Bf00044731.

[ece34622-bib-0046] Koleff, P. , & Gaston, K. J. (2001). Latitudinal gradients in diversity: Real patterns and random models. Ecography, 24, 341–351. 10.1034/j.1600-0587.2001.240312.x

[ece34622-bib-0047] Lennon, J. J. , Koleff, P. , Greenwood, J. J. D. , & Gaston, K. J. (2001). The geographical structure of British bird distributions: Diversity, spatial turnover and scale. Journal of Animal Ecology, 70, 966–979. 10.1046/j.0021-8790.2001.00563.x

[ece34622-bib-0048] Leprieur, F. , Tedesco, P. A. , Hugueny, B. , Beauchard, O. , Durr, H. H. , Brosse, S. , & Oberdorff, T. (2011). Partitioning global patterns of freshwater fish beta diversity reveals contrasting signatures of past climate changes. Ecology Letters, 14, 325–334. 10.1111/j.1461-0248.2011.01589.x. 21303436

[ece34622-bib-0049] Levanoni, O. , Levin, N. , Pe'er, G. , Turbe, A. , & Kark, S. (2011). Can we predict butterfly diversity along an elevation gradient from space? Ecography, 34, 372–383. 10.1111/j.1600-0587.2010.06460.x

[ece34622-bib-0050] Lieberman, D. , Lieberman, M. , Peralta, R. , & Hartshorn, G. S. (1996). Tropical forest structure and composition on a large‐scale altitudinal gradient in Costa Rica. Journal of Ecology, 84, 137–152. 10.2307/2261350

[ece34622-bib-0051] Magurran, A. E. , & McGill, B. J. (2011). Biological diversity: Frontiers in measurement and assessment. Oxford, UK: Oxford University Press.

[ece34622-bib-0053] McCain, C. M. , & Beck, J. (2016). Species turnover in vertebrate communities along elevational gradients is idiosyncratic and unrelated to species richness. Global Ecology and Biogeography, 25, 299–310. 10.1111/geb.12410

[ece34622-bib-0054] Mena, J. L. , & Vazquez‐Dominguez, E. (2005). Species turnover on elevational gradients in small rodents. Global Ecology and Biogeography, 14, 539–547. 10.1111/j.1466-822x.2005.00189.x.

[ece34622-bib-0056] Naniwadekar, R. , & Vasudevan, K. (2007). Patterns in diversity of anurans along an elevational gradient in the Western Ghats, South India. Journal of Biogeography, 34, 842–853. 10.1111/j.1365-2699.2006.01648.x

[ece34622-bib-0057] Navarro, A. G. (1992). Altitudinal Distribution of Birds in the Sierra Madre‐Del‐Sur, Guerrero, Mexico. Condor, 94, 29–39.

[ece34622-bib-0058] Nekola, J. C. , & White, P. S. (1999). The distance decay of similarity in biogeography and ecology. Journal of Biogeography, 26, 867–878. 10.1046/j.1365-2699.1999.00305.x

[ece34622-bib-0059] Oksanen, J. , Kindt, R. , Legendre, P. , O’Hara, B. , Stevens, M. H. H. , Oksanen, M. J. , & Suggests, M. (2007). The Vegan package. Community Ecology Package, 10, 631–637.

[ece34622-bib-0060] O'Malley, M. A. (2008). 'Everything is everywhere: But the environment selects': Ubiquitous distribution and ecological determinism in microbial biogeography. Studies in History & Philosophy of Science Part C Studies in History & Philosophy of Biological & Biomedical Sciences, 39, 314–325. 10.1016/j.shpsc.2008.06.005 18761283

[ece34622-bib-0062] Pan, X. Y. , Ding, Z. F. , Hu, Y. M. , Liang, J. C. , Wu, Y. J. , Si, X. F. , … Jin, K. (2016). Elevational pattern of bird species richness and its causes along a central Himalaya gradient. China. Peerj, 4, ARTNe2636. 10.7717/peerj.2636 PMC5101612 27833806

[ece34622-bib-0063] Paudel, S. , & Vetaas, O. R. (2014). Effects of topography and land use on woody plant species composition and beta diversity in an arid Trans‐Himalayan landscape, Nepal. Journal of Mountain Science, 11, 1112–1122. 10.1007/s11629-013-2858-3

[ece34622-bib-0064] Poulsen, B. O. , Krabbe, N. , Frølander, A. , Hinojosa, M. B. , & Quiroga, C. O. (1997). A rapid assessment of bolivian and ecuadorian montane avifaunas using 20‐species lists: Efficiency, biases and data gathered. Bird Conservation International, 7, 53–67.

[ece34622-bib-0065] Qian, H. , & Ricklefs, R. E. (2007). A latitudinal gradient in large‐scale beta diversity for vascular plants in North America. Ecology Letters, 10, 737–744. 10.1111/j.1461-0248.2007.01066.x 17594429

[ece34622-bib-0066] Qian, H. , Ricklefs, R. E. , & White, P. S. (2005). Beta diversity of angiosperms in temperate floras of eastern Asia and eastern North America. Ecology Letters, 8, 15–22. 10.1111/j.1461-0248.2004.00682.x

[ece34622-bib-0067] Rahbek, C. (1995). The elevational gradient of species richness ‐ a uniform pattern. Ecography, 18, 200–205. 10.1111/j.1600-0587.1995.tb00341.x

[ece34622-bib-0069] Rahbek, C. (2005). The role of spatial scale and the perception of large‐scale species‐richness patterns. Ecology Letters, 8, 224–239. 10.1111/j.1461-0248.2004.00701.x

[ece34622-bib-0070] Rejmanek, M. (2000). Invasive plants: Approaches and predictions. Austral Ecology, 25, 497–506. 10.1046/j.1442-9993.2000.01080.x

[ece34622-bib-0071] Rowe, R. (2009). Environmental and geometric drivers of small mammal diversity along elevational gradients in Utah. Ecography, 32, 411–422. 10.1111/j.1600-0587.2008.05538.x

[ece34622-bib-0072] Saha, S. , Rajwar, G. S. , & Kumar, M. (2016). Forest structure, diversity and regeneration potential along altitudinal gradient in Dhanaulti of Garhwal Himalaya. Forest System, 25, UNSP e058. 10.5424/fs/2016252-07432

[ece34622-bib-0073] Simpson, G. G. (1943). Mammals and the nature of' continents. American Journal of Science, 241, 1–31. 10.2475/ajs.241.1.1

[ece34622-bib-0074] Soininen, J. , Lennon, J. J. , & Hillebrand, H. (2007). A multivariate analysis of beta diversity across organisms and environments. Ecology, 88, 2830–2838. 10.1890/06-1730.1 18051652

[ece34622-bib-0075] Spencer, M. , Schwartz, S. S. , & Blaustein, L. (2002). Are there fine‐scale spatial patterns in community similarity among temporary freshwater pools? Global Ecology and Biogeography, 11, 71–78. 10.1046/j.1466-822X.2001.00266.x

[ece34622-bib-0076] Stevens, G. C. (1992). The elevational gradient in altitudinal range: An extension of Rapoport's latitudinal rule to altitude. American Naturalist, 140, 893–911. 10.1086/285447 19426029

[ece34622-bib-0077] Svenning, J. C. , Flojgaard, C. , & Baselga, A. (2011). Climate, history and neutrality as drivers of mammal beta diversity in Europe: Insights from multiscale deconstruction. Journal of Animal Ecology, 80, 393–402. 10.1111/j.1365-2656.2010.01771.x 21070238

[ece34622-bib-0079] Tuomisto, H. , Ruokolainen, K. , & Yli‐Halla, M. (2003). Dispersal, environment, and floristic variation of western Amazonian forests. Science, 299, 241–244. 10.1126/science.1078037 12522248

[ece34622-bib-0080] Vazquez, G. J. A. , & Givnish, T. J. (1998). Altitudinal gradients in tropical forest composition, structure, and diversity in the Sierra de Manantalan. Journal of Ecology, 86, 999–1020.

[ece34622-bib-0081] Whittaker, R. H. (1975). Communities and ecosystems. New York, NY: Macmillan.

[ece34622-bib-0082] Wilson, M. V. , & Shmida, A. (1984). Measuring beta diversity with presence absence data. Journal of Ecology, 72, 1055–1064. 10.2307/2259551

[ece34622-bib-0083] Winter, M. , Kuhn, I. , La Sorte, F. A. , Schweiger, O. , Nentwig, W. , & Klotz, S. (2010). The role of non‐native plants and vertebrates in defining patterns of compositional dissimilarity within and across continents. Global Ecology and Biogeography, 19, 332–342. 10.1111/j.1466-8238.2010.00520.x

[ece34622-bib-0084] Wolf, J. H. D. (1993). Diversity patterns and biomass of epiphytic bryophytes and lichens along an altitudinal gradient in the Northern Andes. Annals of the Missouri Botanical Garden, 80, 928–960. 10.2307/2399938.

[ece34622-bib-0085] Wu, Y. J. , Colwell, R. K. , Rahbek, C. , Zhang, C. L. , Quan, Q. , Wang, C. K. , & Lei, F. M. (2013). Explaining the species richness of birds along a subtropical elevational gradient in the Hengduan Mountains. Journal of Biogeography, 40, 2310–2323. 10.1111/jbi.12177

[ece34622-bib-0086] Young, B. E. , DeRosier, D. , & Powell, G. V. N. (1998). Diversity and conservation of understory birds in the Tilaran mountains, Costa Rica. The Auk, 115, 998–1016. 10.2307/4089518

[ece34622-bib-0087] Zhang, R. Z. (2011). Zoogeography of China. Beijing, China: Science Press.

[ece34622-bib-0088] Zheng, G. M. (2011). A checklist on the classification and distribution of the birds of China. Beijing, China: Science Press.

