# Corrigendum

**DOI:** 10.1002/ece3.5089

**Published:** 2019-04-16

In “Birds in the Himalayas: What drives beta diversity patterns along an elevational gradient?”, which was published in issue 23, December 2018, the funding information and Acknowledgments were incomplete. The correct information is printed below.

## FUNDING INFORMATION

National Natural Science Foundation of China, Grant/Award Number: 31400361 and 31372175; Ministry of Science and Technology, Grant/Award Number: 2013FY110300; GDAS Special Project of Science and Technology Development (2017GDASCX‐0107).
