# Supplementary material for: Birds in the Himalayas: What drives beta diversity patterns along an elevational gradient?
Source: Ecol Evol. 2018 Nov 8;8(23):11704–16. doi: 10.1002/ece3.4622 (PMC6303779; doi:10.1002/ece3.4622)
Supplement: Supplementary file 1 [file ECE3-8-11704-s001.docx]

**SUPPORTING INFORMATION**

**What drive the beta diversity pattern of birds along a central Himalaya elevational gradient, China?**

**Appendix S1** Additional figure (Fig. S1) and tables (Tables S1, S2).

**Appendix S2** Results for the db-RDA Models the variance partitioning.

**Appendix S1** Additional figure (Fig. S1) and tables (Tables S1–S2).

**Fig. S1** Sample-based rarefaction curves for bird data sets(a-g) for each elevational band in Gyirong valley (“a” is the 300-m elevational band ranges from 1800 m to 2100 m asl.). Observed (circles) and estimated species richness (MMMean statistic: squares; Chao2 statistic: triangles) are expressed as a function of the number of 10-species lists. Sample accumulation order of all curves was randomized 50 times, and each point represents the mean of the resulting 50 values.

**Table S1** Pearson’s correlation matrix for the environmental variables in Gyriong Valley.

|  | MDT | P | PR | HH | Area | MDE | TC |
| --- | --- | --- | --- | --- | --- | --- | --- |
| P | 0.9155** |  |  |  |  |  |  |
| PR | 0.8462** | 0.8869** |  |  |  |  |  |
| HH | -0.0421 | 0.0922 | 0.1243 |  |  |  |  |
| Area | -0.9441** | -0.8295** | -0.7507** | 0.3426 |  |  |  |
| MDE | -0.0439 | 0.0806 | 0.1408 | 0.9569** | 0.3389 |  |  |
| TC | 0.6703* | 0.4921 | 0.4784 | -0.3915 | -0.7453* | -0.3640 |  |
| PC | 0.7853 | 0.5892* | 0.5089 | -0.4787 | -0.8710** | -0.5007 | 0.8508** |

“*”: *P*<0.05, *P*<0.01; MDT: mean daily temperature; P: Precipitation; HH: habitat heterogeneity; MDE: mid domain effect; TC: Temperature change between present and the LGM. Negative relationships are indicated by “-”.

**Table S2** Species checklists of all birds recorded over survey period in the Gyirong Valley.

| Order | Family | Scientific name | Breeding | Endemic |
| --- | --- | --- | --- | --- |
| Anseriformes | Anatidae | *Anas penelope* |  |  |
| Anseriformes | Anatidae | *Aythya fuligula* |  |  |
| Falconiformes | Accipitridae | *Accipiter badius* | B |  |
| Falconiformes | Accipitridae | *Accipiter trivirgatus* | B |  |
| Falconiformes | Accipitridae | *Aquila chrysaetos* | B |  |
| Falconiformes | Accipitridae | *Aqula clanga* |  |  |
| Falconiformes | Accipitridae | *Aquila nipalensis* | B |  |
| Falconiformes | Accipitridae | *Buteo buteo* | B |  |
| Falconiformes | Accipitridae | *Buteo hemilasius* | B |  |
| Falconiformes | Accipitridae | *Circus cyaneus* |  |  |
| Falconiformes | Accipitridae | *Gypaetus barbatus* | B |  |
| Falconiformes | Accipitridae | *Gyps fulvus* | B |  |
| Falconiformes | Accipitridae | *Gyps himalayensis* | B |  |
| Falconiformes | Accipitridae | *Milvus migrans* | B |  |
| Falconiformes | Accipitridae | *Pernis ptilorhyncus* |  |  |
| Falconiformes | Falconidae | *Falco subbuteo* | B |  |
| Falconiformes | Falconidae | *Falco tinnunculus* | B |  |
| Galliformes | Phasianidae | *Alectoris chukar* | B |  |
| Galliformes | Phasianidae | *Ithaginis cruentus* | B | E |
| Galliformes | Phasianidae | *Lophophorus impejanus* | B | E |
| Galliformes | Phasianidae | *Lophura leucomelanos* | B |  |
| Galliformes | Phasianidae | *Perdix hodgsoniae* | B | E |
| Galliformes | Phasianidae | *Tetraogallus tibetanus* | B |  |
| Gruiformes | Rallidae | *Gallinula chloropus* | B |  |
| Charadriiformes | Ibidorhynchidae | *Ibidorhyncha struthersii* | B |  |
| Charadriiformes | Rostratulidae | *Rostratula benghalensis* | B |  |
| Charadriiformes | Scolopacidae | *Actitis hypoleucos* |  |  |
| Columbiformes | Columbidae | *Columba hodgsonii* | B | E |
| Columbiformes | Columbidae | *Columba leuconota* | B | E |
| Columbiformes | Columbidae | *Columba livia* | B |  |
| Columbiformes | Columbidae | *Columba rupestris* | B |  |
| Columbiformes | Columbidae | *Streptopelia orientalis* | B |  |
| Columbiformes | Columbidae | *Streptopelia turtur* | B |  |
| Columbiformes | Columbidae | *Treron sphenurus* | B |  |
| Cuculiformes | cuculidae | *Cuculus micropterus* | B |  |
| Cuculiformes | cuculidae | *Cuculus sparverioides* | B |  |
| Strigiformes | Strigidae | *Athene noctua* | B |  |
| Strigiformes | Strigidae | *Glaucidium brodiei* | B |  |
| Apodiformes | Apodidae | *Apus nipalensis* | B |  |
| Upupiformes | Upupidae | *Upupa epops* | B |  |
| Piciformes | Capitonidae | *Megalaima virens* | B |  |
| Piciformes | Picidae | *Dendrocopos auriceps* | B |  |
| Piciformes | Picidae | *Dendrocopos cathpharius* | B | E |
| Piciformes | Picidae | *Dendrocopos darjellensis* | B | E |
| Piciformes | Picidae | *Jynx torquilla* | B |  |
| Piciformes | Picidae | *Picus squamatus* | B |  |
| Passeriformes | Alaudidae | *Alauda gulgula* | B |  |
| Passeriformes | Alaudidae | *Calandrella cheleensis* | B |  |
| Passeriformes | Alaudidae | *Eremophila alpestris* | B |  |
| Passeriformes | Hirundinidae | *Hirundo rustica* | B |  |
| Passeriformes | Hirundinidae | *Ptyonoprogne rupestris* | B |  |
| Passeriformes | Hirundinidae | *Riparia riparia* | B |  |
| Passeriformes | Motacillidae | *Anthus hodgsoni* |  |  |
| Passeriformes | Motacillidae | *Anthus roseatus* | B | E |
| Passeriformes | Motacillidae | *Motacilla alba* | B |  |
| Passeriformes | Motacillidae | *Motacilla cinerea* |  |  |
| Passeriformes | Motacillidae | *Motacilla citreola* | B |  |
| Passeriformes | Motacillidae | *Motacilla flava* |  |  |
| Passeriformes | Campephagidae | *Pericrocotus brevirostris* | B | E |
| Passeriformes | Campephagidae | *Pericrocotus ethologus* | B | E |
| Passeriformes | Pycnonotidae | *Hypsipetes leucocephalus* | B |  |
| Passeriformes | Pycnonotidae | *Pycnonotus jocosus* | B |  |
| Passeriformes | Pycnonotidae | *Pycnonotus leucogenys* | B |  |
| Passeriformes | Laniidae | *Lanius tephronotus* | B | E |
| Passeriformes | Oriolidae | *Oriolus traillii* | B |  |
| Passeriformes | Dicruridae | *Dicrurus macrocercus* | B |  |
| Passeriformes | Corvidae | *Cissa chinensis* | B |  |
| Passeriformes | Corvidae | *Corvus corax* | B |  |
| Passeriformes | Corvidae | *Corvus macrorhynchos* | B |  |
| Passeriformes | Corvidae | *Nucifraga caryocatactes* | B |  |
| Passeriformes | Corvidae | *Pyrrhocorax graculus* | B |  |
| Passeriformes | Corvidae | *Pyrrhocorax pyrrhocorax* | B |  |
| Passeriformes | Corvidae | *Urocissa flavirostris* | B | E |
| Passeriformes | Cinclidae | *Cinclus cinclus* | B |  |
| Passeriformes | Cinclidae | *Cinclus pallasii* | B |  |
| Passeriformes | Troglodytidae | *Troglodytes troglodytes* | B |  |
| Passeriformes | Prunellidae | *Prunella fulvescens* | B |  |
| Passeriformes | Prunellidae | *Prunella rubeculoides* | B |  |
| Passeriformes | Prunellidae | *Prunella strophiata* | B | E |
| Passeriformes | Turdidae | *Chaimarrornis leucocephalus* | B | E |
| Passeriformes | Turdidae | *Enicurus scouleri* | B |  |
| Passeriformes | Turdidae | *Grandala coelicolor* | B | E |
| Passeriformes | Turdidae | *Hodgsonius phoenicuroides* | B | E |
| Passeriformes | Turdidae | *Luscinia brunnea* | B | E |
| Passeriformes | Turdidae | *Luscinia pectoralis* | B | E |
| Passeriformes | Turdidae | *Monticola rufiventris* | B |  |
| Passeriformes | Turdidae | *Monticola solitarius* | B |  |
| Passeriformes | Turdidae | *Myophonus caeruleus* | B |  |
| Passeriformes | Turdidae | *Oenanthe deserti* | B |  |
| Passeriformes | Turdidae | *Phoenicurus erythrogaster* | B |  |
| Passeriformes | Turdidae | *Phoenicurus frontalis* | B | E |
| Passeriformes | Turdidae | *Phoenicurus ochruros* | B |  |
| Passeriformes | Turdidae | *Rhyacornis fuliginosus* | B |  |
| Passeriformes | Turdidae | *Saxicola ferreus* | B |  |
| Passeriformes | Turdidae | *Saxicola torquata* | B |  |
| Passeriformes | Turdidae | *Tarsiger chrysaeus* | B | E |
| Passeriformes | Turdidae | *Tarsiger cyanurus* | B |  |
| Passeriformes | Turdidae | *Tarsiger indicus* | B | E |
| Passeriformes | Turdidae | *Turdus albocinctus* | B | E |
| Passeriformes | Turdidae | *Turdus merula* | B |  |
| Passeriformes | Turdidae | *Turdus ruficollis* |  |  |
| Passeriformes | Muscicapidae | *Culicicapa ceylonensis* | B |  |
| Passeriformes | Muscicapidae | *Eumyias thalassina* | B |  |
| Passeriformes | Muscicapidae | *Ficedula hyperythra* | B | E |
| Passeriformes | Muscicapidae | *Ficedula strophiata* | B |  |
| Passeriformes | Muscicapidae | *Ficedula superciliaris* | B |  |
| Passeriformes | Muscicapidae | *Ficedula tricolor* | B | E |
| Passeriformes | Muscicapidae | *Muscicapa dauurica* | B |  |
| Passeriformes | Muscicapidae | *Muscicapa sibirica* | B |  |
| Passeriformes | Muscicapidae | *Niltava sundara* | B | E |
| Passeriformes | Rhipiduridae | *Rhipidura hypoxantha* | B | E |
| Passeriformes | Timaliidae | *Alcippe vinipectus* | B | E |
| Passeriformes | Timaliidae | *Garrulax affinis* | B | E |
| Passeriformes | Timaliidae | *Garrulax erythrocephalus* | B | E |
| Passeriformes | Timaliidae | *Garrulax lineatus* | B | E |
| Passeriformes | Timaliidae | *Garrulax ocellatus* | B | E |
| Passeriformes | Timaliidae | *Garrulax striatus* | B | E |
| Passeriformes | Timaliidae | *Garrulax variegatus* | B | E |
| Passeriformes | Timaliidae | *Heterophasia capistrata* | B | E |
| Passeriformes | Timaliidae | *Minla strigula* | B | E |
| Passeriformes | Timaliidae | *Pnoepyga albiventer* | B | E |
| Passeriformes | Timaliidae | *Pteruthius xanthochlorus* | B | E |
| Passeriformes | Timaliidae | *Yuhina gularis* | B | E |
| Passeriformes | Timaliidae | *Yuhina flavicollis* | B | E |
| Passeriformes | Timaliidae | *Yuhina occipitalis* | B | E |
| Passeriformes | Cisticolidae | *Prinia criniger* | B |  |
| Passeriformes | Cisticolidae | *Prinia hodgsonii* | B |  |
| Passeriformes | Sylviidae | *Cettia brunnifrons* | B | E |
| Passeriformes | Sylviidae | *Cettia flavolivaceus* | B | E |
| Passeriformes | Sylviidae | *Cettia major* | B | E |
| Passeriformes | Sylviidae | *Cettia pallidipes* | B |  |
| Passeriformes | Sylviidae | *Phylloscopus affinis* | B | E |
| Passeriformes | Sylviidae | *Phylloscopus borealis* |  |  |
| Passeriformes | Sylviidae | *Phylloscopus chloronotus* |  |  |
| Passeriformes | Sylviidae | *Phylloscopus fuscatus* | B |  |
| Passeriformes | Sylviidae | *Phylloscopus humei* | B |  |
| Passeriformes | Sylviidae | *Phylloscopus inornatus* |  |  |
| Passeriformes | Sylviidae | *Phylloscopus maculipennis* | B | E |
| Passeriformes | Sylviidae | *Phylloscopus magnirostris* | B | E |
| Passeriformes | Sylviidae | *Phylloscopus pulcher* | B | E |
| Passeriformes | Sylviidae | *Phylloscopus reguloides* | B |  |
| Passeriformes | Sylviidae | *Phylloscopus trochiloides* | B |  |
| Passeriformes | Sylviidae | *Seicercus burkii* | B |  |
| Passeriformes | Sylviidae | *Seicercus xanthoschistos* | B | E |
| Passeriformes | Sylviidae | *Tesia castaneocoronata* | B | E |
| Passeriformes | Zosteropidae | *Zosterops japonicus* | B |  |
| Passeriformes | Aegithalidae | *Aegithalos concinnus* | B |  |
| Passeriformes | Aegithalidae | *Aegithalos iouschistos* | B | E |
| Passeriformes | Paridae | *Parus ater* | B |  |
| Passeriformes | Paridae | *Parus dichrous* | B | E |
| Passeriformes | Paridae | *Parus major* | B |  |
| Passeriformes | Paridae | *Parus monticolus* | B |  |
| Passeriformes | Paridae | *Parus rubidiventris* | B | E |
| Passeriformes | Paridae | *Pseudopodoces humilis* | B |  |
| Passeriformes | Sittidae | *Sitta himalayensis* | B | E |
| Passeriformes | Trichodoninae | *Tichodroma muraria* | B |  |
| Passeriformes | Certhiidae | *Certhia familiaris* | B |  |
| Passeriformes | Certhiidae | *Certhia nipalensis* | B | E |
| Passeriformes | Dicaeidae | *Dicaeum ignipectus* | B |  |
| Passeriformes | Bombycillidae | *Aethopyga gouldiae* | B |  |
| Passeriformes | Bombycillidae | *Aethopyga ignicauda* | B | E |
| Passeriformes | Bombycillidae | *Aethopyga nipalensis* | B | E |
| Passeriformes | Passeridae | *Montifringilla adamsi* | B |  |
| Passeriformes | Passeridae | *Passer montanus* | B |  |
| Passeriformes | Frigillidae | *Carpodacus edwardsii* | B | E |
| Passeriformes | Frigillidae | *Carpodacus erythrinus* | B |  |
| Passeriformes | Frigillidae | *Carpodacus nipalensis* | B | E |
| Passeriformes | Frigillidae | *Carpodacus pulcherrimus* | B | E |
| Passeriformes | Frigillidae | *Carpodacus puniceus* | B |  |
| Passeriformes | Frigillidae | *Carpodacus rhodopeplus* | B | E |
| Passeriformes | Frigillidae | *Carpodacus rodochroa* | B | E |
| Passeriformes | Frigillidae | *Carpodacus rubicilla* | B |  |
| Passeriformes | Frigillidae | *Carpodacus rubicilloides* | B |  |
| Passeriformes | Frigillidae | *Carpodacus thura* | B | E |
| Passeriformes | Frigillidae | *Carduelis flavirostris* | B |  |
| Passeriformes | Frigillidae | *Carduelis spinoides* | B |  |
| Passeriformes | Frigillidae | *Haematospiza sipahi* | B | E |
| Passeriformes | Frigillidae | *Leucosticte brandti* | B |  |
| Passeriformes | Frigillidae | *Mycerobas carnipes* | B |  |
| Passeriformes | Frigillidae | *Pinicola subhimachala* | B | E |
| Passeriformes | Frigillidae | *Pyrrhula erythrocephala* | B | E |
| Passeriformes | Frigillidae | *Serinus pusillus* | B |  |

Endemic species were defined based on their distributions limited to the Himalayas and the western Hengduan Mountains.

**Appendix S2** Results for the db-RDA Models and the variance partitioning.

## S2.1 db-RDA.all

Inertia Proportion Eigenvals Rank

Total 1.977252 1.000000 2.104004

Constrained 1.964344 0.993472 2.044103 8

Unconstrained 0.012908 0.006528 0.059902 3

Imaginary -0.126752 3

Inertia is squared Beta.sim distance

Eigenvalues for constrained axes:

CAP1 CAP2 CAP3 CAP4 CAP5 CAP6 CAP7 CAP8

1.7254 0.2211 0.0688 0.0220 0.0031 0.0022 0.0014 0.0001

Eigenvalues for unconstrained axes:

MDS1 MDS2 MDS3

0.03755 0.01822 0.00413

> ef.all

***VECTORS

CAP1 CAP2 r2 Pr(>r)

MDT -0.99016 0.13997 0.9654 0.001 ***

P -0.98786 -0.15533 0.9844 0.001 ***

PR -0.99621 -0.08703 0.8025 0.001 ***

HH -0.02410 -0.99971 0.5609 0.023 *

Area 0.94189 -0.33592 0.9129 0.001 ***

MDE -0.01061 -0.99994 0.5927 0.022 *

TC -0.82902 0.55922 0.4999 0.038 *

PC -0.79779 0.60293 0.8080 0.002 **

---

Signif. codes: 0 ‘***’ 0.001 ‘**’ 0.01 ‘*’ 0.05 ‘.’ 0.1 ‘ ’ 1

Permutation: free

Number of permutations: 999

## S2.2 dbrda.select

Inertia Proportion Eigenvals Rank

Total 1.97725 1.00000 2.10400

Constrained 1.90474 0.96333 1.95763 5

Unconstrained 0.07251 0.03667 0.14638 6

Imaginary -0.12675 3

Inertia is squared Beta.sim distance

Eigenvalues for constrained axes:

CAP1 CAP2 CAP3 CAP4 CAP5

1.7029 0.1852 0.0622 0.0057 0.0016

Eigenvalues for unconstrained axes:

MDS1 MDS2 MDS3 MDS4 MDS5 MDS6

0.07174 0.03384 0.01862 0.01554 0.00543 0.00120

> ef.select

***VECTORS

CAP1 CAP2 r2 Pr(>r)

P -0.99046 0.13783 0.9848 0.001 ***

PR -0.99731 0.07328 0.8024 0.002 **

Area 0.94602 0.32411 0.9145 0.001 ***

MDE -0.06402 0.99795 0.5831 0.034 *

TC -0.83587 -0.54892 0.5040 0.051 .

---

Signif. codes: 0 ‘***’ 0.001 ‘**’ 0.01 ‘*’ 0.05 ‘.’ 0.1 ‘ ’ 1

Permutation: free

Number of permutations: 999

## S2.3 db-RDA.step

Inertia Proportion Eigenvals Rank

Total 1.97725 1.00000 2.10400

Constrained 1.86265 0.94204 1.86774 2

Unconstrained 0.11460 0.05796 0.23627 8

Imaginary -0.12675 3

Inertia is squared Beta.sim distance

Eigenvalues for constrained axes:

CAP1 CAP2

1.6957 0.1720

Eigenvalues for unconstrained axes:

MDS1 MDS2 MDS3 MDS4 MDS5 MDS6 MDS7 MDS8

0.09460 0.07748 0.03128 0.01917 0.00795 0.00381 0.00177 0.00019

> ef.step

***VECTORS

CAP1 CAP2 r2 Pr(>r)

P -0.99032 0.13881 0.9867 0.001 ***

Area 0.94943 0.31398 0.9150 0.001 ***

---

Signif. codes: 0 ‘***’ 0.001 ‘**’ 0.01 ‘*’ 0.05 ‘.’ 0.1 ‘ ’ 1

Permutation: free

Number of permutations: 999
